# Burden and temporal trends of female-specific cancers in China: A systematic analysis of the 2023 global burden of disease study

**DOI:** 10.1371/journal.pone.0351539

**Published:** 2026-06-10

**Authors:** Shaoxing Chen, Xiaohuang Yang, Shaona Jiang, Yina Lin, Leijuan Huang, Huiqin Chen, Jinzhu Zheng, Yuanfu Xie, Zhixing Kuang

**Affiliations:** 1 Department of Radiation Oncology, Zhangzhou Affiliated Hospital of Fujian Medical University, Zhangzhou, Fujian, China; 2 Department of Gastroenterology, Zhangzhou Affiliated Hospital of Fujian Medical University, Zhangzhou, Fujian, China; 3 Department of Health Management Center, Zhangzhou Affiliated Hospital of Fujian Medical University, Zhangzhou, Fujian, China; 4 Department of Radiotherapy, Fujian Medical University Union Hospital, Fuzhou, Fujian, China; Lorestan University, IRAN, ISLAMIC REPUBLIC OF

## Abstract

**Background:**

Female-specific cancers represent a substantial public health challenge in China. This study was designed to evaluate temporal trends and generate future projections of the burden associated with female-specific cancers in China from 1990 to 2023.

**Methods:**

Data on incidence, mortality, and disability-adjusted life-years (DALYs) attributable to breast, cervical, uterine, and ovarian cancers among women in China were extracted from the Global Burden of Disease (GBD) 2023 database. Joinpoint analysis was used to assess temporal trends, whereas decomposition analysis was applied to quantify the contributions of epidemiological changes, population growth, and ageing to changes in disease burden; a Bayesian age-period-cohort (BAPC) model was further used to project trends in disease burden over the next 15 years.

**Results:**

Between 1990 and 2023, all age-standardized rates (ASRs) of female-specific cancers in China declined except for the age-standardized incidence rate (ASIR) of breast cancer, which increased from 24.54 (95% uncertainty interval [UI]: 19.7–30.79) per 100,000 in 1990 to 31.62 (95% UI: 25.4–38.22) per 100,000 in 2023. The absolute disease burden increased, with 342,725 (95% UI: 270,872–413,171) new breast cancer cases in 2023, representing a 187.24% increase. In contrast, the numbers of new cases of cervical, uterine, and ovarian cancers were 124,686 (95% UI: 77,638–165,722), 74,990 (95% UI: 53,630–119,885), and 38,969 (95% UI: 31,914–46,805), respectively, with corresponding increases of 43.63%, 82.97%, and 61.76%. Population aging and growth were the main contributing factors, and the BAPC model projected that the absolute disease burden of all four female-specific cancers would continue to increase in the future.

**Conclusion:**

The burden of female-specific cancers in China remains substantial, underscoring the urgent need to develop targeted prevention and intervention strategies to mitigate this burden.

## Introduction

Global cancer statistics indicate that breast cancer is the most common malignancy among women, with approximately 2,295,700 new cases worldwide in 2022, accounting for 23.8% of all female cancers [1]. Cervical, uterine, and ovarian cancers are also among the most common malignancies in women; in 2022, there were approximately 661,000 new cases of cervical cancer, 420,200 of uterine cancer, and 324,400 of ovarian cancer worldwide [1]. These four malignancies also pose a significant public health challenge to women in China. Estimates by the National Cancer Center of China suggest that in 2022, the number of newly diagnosed cases of breast, cervical, uterine, and ovarian cancers among women in China was approximately 357,200, 150,700, 77,700, and 61,100, respectively, and breast cancer was the second most common malignancy among women in China, after lung cancer [2]. In addition to threatening women’s lives, these malignancies can impair fertility, exerting both physical and psychological harm on affected women.

The occurrence and progression of breast cancer and gynecological cancers are influenced by multiple factors, including environmental exposures, genetic predisposition, reproductive and hormonal factors (such as early menarche, late menopause, and elevated estrogen levels), unsafe sexual behavior, obesity, human papillomavirus (HPV) infection, and unhealthy lifestyle habits (e.g., alcohol consumption and smoking) [3–14]. Developing targeted risk factor control and effective primary prevention strategies are key steps in reducing the burden of female-specific cancers. Accordingly, the World Health Organization (WHO) launched the Global Strategy to Accelerate the Elimination of Cervical Cancer in 2020, which aims to reduce the global cervical cancer incidence to below 4 per 100,000 women by 2030 through early screening and widespread HPV vaccination; in 2021, it launched the Global Breast Cancer Initiative, designed to reduce global breast cancer mortality through early diagnosis and comprehensive cancer care [15,16]. Furthermore, with advances in modern medicine, substantial progress has been made in the treatment of breast and gynecological cancers—including radiotherapy, surgery, chemotherapy, targeted therapy, immunotherapy, and endocrine therapy—substantially improving patient prognosis [5,17]. As the world’s most populous country, China, with its rapidly aging population, carries a substantial proportion of the global burden of female-specific cancers. Current epidemiological studies on these cancers in China are largely based on the GBD 2021 database [18,19], yet are limited by a lack of updated, population-level epidemiological data. Updated analyses of temporal trends in female-specific cancers in China are critical for the rational allocation of medical resources and the formulation of evidence-based public health policies.

Using data from the GBD 2023 database, this study systematically analyzed the burden of breast, cervical, uterine, and ovarian cancers among women in China, evaluating temporal trends in incidence, mortality, and disability-adjusted life-years (DALYs) and projecting future trends in disease burden. It aimed to provide scientific evidence to inform targeted public health policies for prevention, screening, treatment, and management, thereby promoting the health of women in China.

## Methods

### Data sources

The GBD 2023 study, led by the Institute for Health Metrics and Evaluation (IHME) at the University of Washington, quantified the burden of 375 diseases and injuries as well as 88 risk factors across 204 countries and territories worldwide, providing comprehensive estimates of health loss from 1990 to 2023 and highlighting disease burden trends before and after the COVID-19 pandemic [20]. To adjust for heterogeneous age structures across populations in different regions, the GBD 2023 study applied the GBD 2023 world standard population (S1 Table) to calculate age-standardized rates (ASRs), facilitating cross-regional comparisons of disease burden [21]. The GBD 2023 world standard population is available in the supplementary material of a GBD publication in The Lancet [22]. Data sources for cancer in China within the GBD 2023 framework included the National Mortality Surveillance System (NMSS), cancer registries, population censuses, vital registries, maternal and child surveillance data, cause-of-death data from the Hong Kong Special Administrative Region and the Macao Special Administrative Region, and other published studies and reports [23,24]. Within the GBD 2023 framework, cancer mortality estimates were generated using Cause of Death Ensemble Models (CODEm), which drew on vital registration and verbal autopsy mortality data, along with cancer registry incidence data, which were converted into mortality estimates using mortality-to-incidence ratios (MIRs) [25]. Mortality estimates for cancers were further converted into incidence estimates via MIRs specific to each cancer cause [25]. Years of Life Lost (YLLs) were estimated as the product of the number of cancer deaths in each age group and the standard life expectancy for that age group; Years Lived with Disability (YLDs) were calculated as the product of the prevalence of each cancer stage and the stage-specific disability weight; DALYs were the sum of YLLs and YLDs(25).To quantify uncertainty in estimates, the GBD 2023 study computed 95% uncertainty intervals (UIs) for each indicator as the 2.5th and 97.5th percentiles from 250 draws of the distribution of each metric [26]. In the present study, we downloaded data on incidence, mortality, DALYs, and corresponding 95% UIs for female-specific cancers—including breast, cervical, uterine, and ovarian cancers—in China from the Global Health Data Exchange (GHDx) query tool (https://vizhub.healthdata.org/gbd-results/). This study is a secondary analysis of the GBD 2023 database, conducted in strict accordance with the data use policies for GBD 2023 established by IHME, and therefore required no ethical approval or informed consent.

### Case definition

The International Classification of Diseases, 10th Revision (ICD-10) codes for breast, cervical, uterine, and ovarian cancers are presented in S2 Table.

### Statistical analysis

Joinpoint regression analysis was applied to evaluate temporal trends in the age-standardized incidence rate (ASIR), age-standardized mortality rate (ASMR), and age-standardized DALY rate (ASDR) of female-specific cancers in China from 1990 to 2023. This approach enables the identification of specific years when significant changes occur in disease burden trends, allowing for precise characterization of the dynamic patterns underlying variations in burden. Segmented regression models were optimized via the grid search method (GSM) to minimize mean squared error and validated using Monte Carlo permutation tests, with a maximum of 5 joinpoints identified [27]. Annual percentage change (APC) was estimated using log-linear models to characterize disease burden trends within specific time intervals. The average annual percentage change (AAPC), representing the overall trend from 1990 to 2023, was derived as the weighted average of APCs obtained from the joinpoint models [28]. The formulas for APC and AAPC are as follows:


$$\begin{array}{c} \text{AP}{\text{C}}_{\text{i}}=\left(e^{b_{i}}-1\right)\text{x}100 \end{array}$$

$$\begin{array}{c} \text{AAPC}=({\text{e}}^{\sum w_{i}b_{i}/\sum w_{i}}-\text{l})\text{x}100 \end{array}$$

(APCi: The annual percent change in the i-th segment; $b_{i}$: The slope coefficient of the log-linear regression model for the i-th segment; $w_{i}$: The weight of the i-th segment, defined as the number of years covered by the segment.)

Temporal trends in ASRs of female-specific cancers in China were evaluated using the AAPC. Statistical significance of the AAPC was assessed using both 95% confidence intervals (CIs) provided by Joinpoint software and two-sided Z-test P values. A significant increasing trend was defined as a 95% CI entirely above 0 with P < 0.05; a significant decreasing trend was defined as a 95% CI entirely below 0 with P < 0.05; and no statistically significant trend was identified if the 95% CI included 0 with P ≥ 0.05.

An Age-Period-Cohort model was used to disentangle temporal trends into age, period, and cohort effects, and to evaluate their independent impacts on the incidence, mortality, and DALYs of female-specific cancers in China [29]. Parameter estimates were obtained using the Age-Period-Cohort web tool developed by the USA National Cancer Institute (https://analysistools.cancer.gov/apc/) [30]. In this study, age was categorized into 17 5-year age groups (15–19–95 + years) for breast, cervical, and ovarian cancers, and into 16 5-year age groups (20–24–95 + years) for uterine cancer. The study period was divided into 6 intervals spanning from 1994–1998–2019–2023. For birth cohorts, 22 overlapping 10-year cohorts (1894–1903–1999–2008) were defined for breast, cervical, and ovarian cancers, while 21 such cohorts (1894–1903–1994–2003) were used for uterine cancer. The mid-period (2004–2008) and mid-birth cohort (1944–1953) served as reference groups. Net drift and local drift represented the overall log-linear trend across all ages and age-specific log-linear trends, respectively [31].

Using the decomposition method developed by Das Gupta, this study quantified the contributions of epidemiological changes, population aging, and population growth to changes in the disease burden of female-specific cancers in China [32]. This approach allows the variation in disease burden between two time points (1990 and 2023) to be decomposed into independent contributions attributable to population aging, epidemiological shifts, and population growth, thereby offering clearer insights into the key drivers underlying these changes.

The BAPC model was applied to forecast the burden of female-specific cancers in China over the next 15 years, providing a scientific basis for targeted prevention and control. Building on the conventional age-period-cohort framework, this model incorporates Bayesian statistical methods and integrated nested Laplace approximation (INLA) to capture temporal trends across age, period, and cohort, thereby enhancing the accuracy of projections [33,34].

This study used R 4.4.2 and Joinpoint Software (v5.1.0) for data analysis and graphical presentation, with P < 0.05 indicating statistical significance.

## Results

### Burden of female-specific cancers in China, 1990–2023

**Fig 1:**
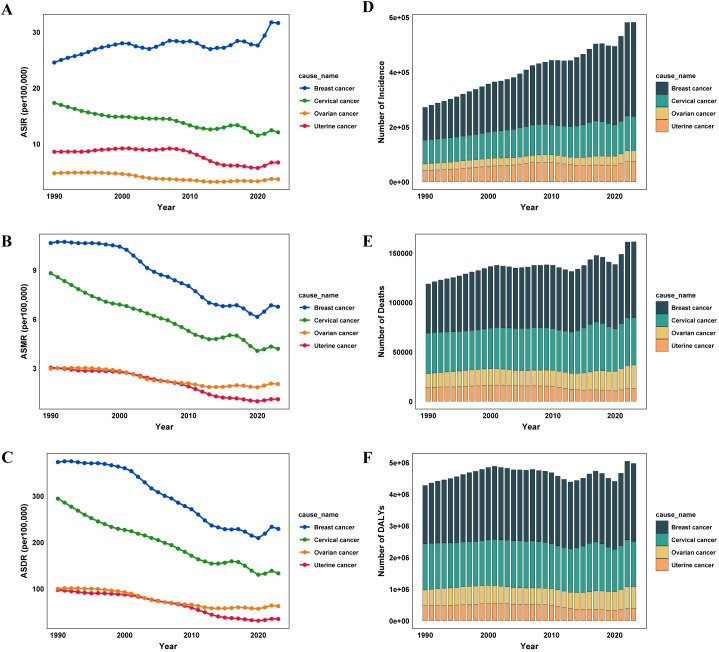
Temporal trends in the burden of female-specific cancers in China, 1990–2023. (A) Age-standardized incidence rates; (B) Age-standardized mortality rates;(C) Age-standardized DALY rates; (D) Number of Incidence;(E) Number of Deaths;(F) Number of DALYs. DALY, disability-adjusted life year‌‌.

From 1990 to 2023, the ASIRs of cervical, uterine, and ovarian cancer in China all showed a downward trend; in contrast, the ASIR of breast cancer exhibited an upward trend, rising from 24.54 per 100,000 (95% UI: 19.7–30.79) in 1990 to 31.62 per 100,000 (95% UI: 25.4–38.22) in 2023 (Fig 1A, Table 1). For all four female-specific cancers, the ASMRs and ASDRs declined between 1990 and 2023, with breast cancer and cervical cancer showing a more substantial decrease, while ovarian cancer and uterine cancer showed a slow downward trend. Throughout the study period, the ASMRs and ASDRs of breast and cervical cancer remained consistently higher than those of uterine and ovarian cancer (Fig 1B-C).In terms of absolute burden, the total number of incident cases, deaths, and DALYs attributable to female-specific cancers in China showed an upward trend between 1990 and 2023; notably, the absolute burden of breast and cervical cancer was substantially higher than that of uterine and ovarian cancer across the study period (Fig 1D-F). The number of new breast cancer cases rose from 119,317 (95% UI: 95,337–149,295) in 1990–342,725 (95% UI: 270,872–413,171) in 2023, an increase of 187.24%; the number of deaths increased from 49,616 (95% UI: 40,599–62,433) in 1990–76,736 (95% UI: 64,152–90,833) in 2023, a rise of 54.66%; and the number of DALYs increased from 1,844,963 (95% UI: 1,493,992–2,303,644) to 2,463,392 (95% UI: 2,082,006–2,920,776) in 2023, a growth of 33.52%. For cervical cancer, the number of new cases and deaths increased by 43.63% and 16.54%, respectively, while DALYs decreased by 1.62% (Table 1).

**Table 1:** Comparison of the burden of female-specific cancers in China, 1990 and 2023.

Measure	Causes	1990		2023	
		Counts	Age-standardized rates per 100,000 people	Counts	Age-standardized rates per 100,000 people
		n (95% UI)	n (95% UI)	n (95% UI)	n (95% UI)
Incidence	Breast cancer	119317 (95337-149295)	24.54 (19.7-30.79)	342725 (270872-413171)	31.62 (25.4-38.22)
Incidence	Cervical cancer	86808 (62053-125564)	17.32 (12.51-25.01)	124686 (77638-165722)	12.06 (7.44-15.96)
Incidence	Ovarian cancer	24091 (18197-30774)	4.73 (3.58-6.07)	38969 (31914-46805)	3.66 (3.05-4.38)
Incidence	Uterine cancer	40984 (24818-53697)	8.58 (5.18-11.28)	74990 (53630-119885)	6.66 (4.75-10.71)
Deaths	Breast cancer	49616 (40599-62433)	10.65 (8.76-13.51)	76736 (64152-90833)	6.76 (5.73-8.03)
Deaths	Cervical cancer	41076 (30145-59132)	8.8 (6.45-12.75)	47872 (30050-62786)	4.19 (2.67-5.48)
Deaths	Ovarian cancer	13997 (10410-18305)	2.98 (2.22-3.9)	23670 (19076-28752)	2.04 (1.67-2.48)
Deaths	Uterine cancer	13940 (8373-18256)	3.04 (1.84-4)	12989 (9769-19901)	1.11 (0.83-1.7)
DALYs	Breast cancer	1844963 (1493992-2303644)	372.92 (301.56-465.93)	2463392 (2082006-2920776)	228.95 (195.62-269.55)
DALYs	Cervical cancer	1457115 (1070025-2064945)	294.14 (215.88-420.49)	1433468 (899335-1881023)	133.06 (83.97-173.65)
DALYs	Ovarian cancer	501308 (370954-644605)	99.83 (74.23-129.35)	686054 (559503-829349)	62.35 (51.81-75.23)
DALYs	Uterine cancer	471077 (284518-612096)	97.12 (58.59-125.92)	387575 (291344-592429)	34.64 (26.1-52.88)

DALY, disability-adjusted life year.

### Age-specific burden of female-specific cancers in China

**Fig 2:**
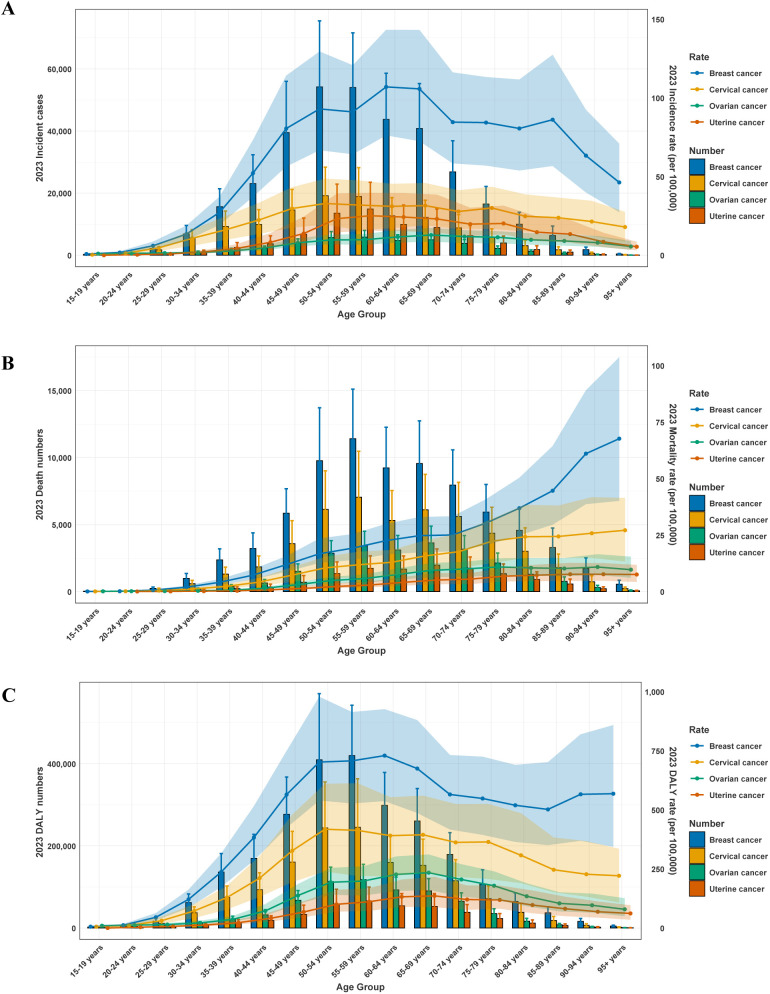
Case numbers and rates of female-specific cancers by age in China, 2023. (A) Incidence;(B) Deaths;(C) DALYs. Error bars and shaded regions denote the 95% uncertainty intervals. DALY, disability-adjusted life year.

**Fig 3:**
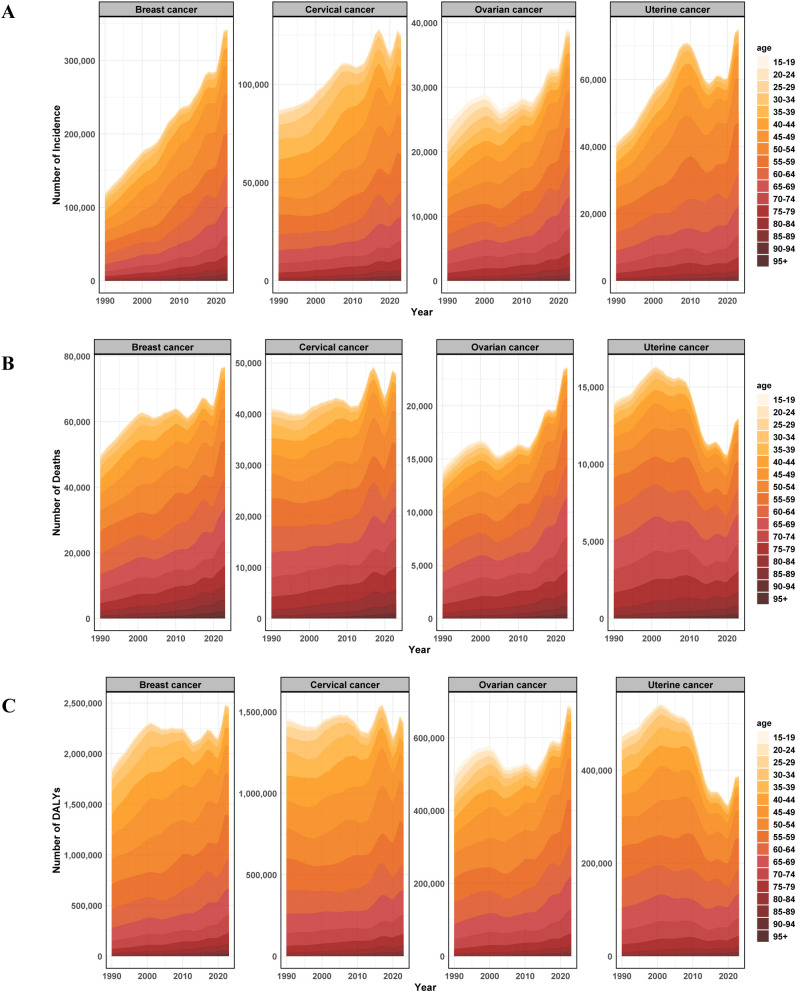
Temporal trends in absolute burden of female-specific cancers by age group in China, 1990–2023. (A) Incident cases; (B) Death numbers; (C) DALY numbers. DALY, disability-adjusted life year.

In 1990, the age-specific incidence rate of cervical, uterine, and ovarian cancers in China showed an initial increase followed by a decline with advancing age; the incidence peaks for cervical and uterine cancers occurred at 65–69 years, while that for ovarian cancer occurred at 55–59 years. By contrast, the age-specific incidence rate of breast cancer generally increased with age, peaking at 90–94 years (S1A Fig). During the same period, the mortality rates and DALY rates of the four female-specific cancers across all age groups exhibited a trend of first rising and then falling, with peaks between 50–54 years and 60–64 years (S1B-C Fig). In 1990, the number of new cases, deaths, and DALYs attributable to female-specific cancers in China across all age groups also exhibited a trend of first increasing and then decreasing (S1 Fig). Compared with 1990, the age-specific relative burden of female-specific cancers in China showed significant differences in 2023, with the differences primarily observed in breast and cervical cancer; the age-specific incidence rate of the former first increased and then decreased, with the peak at 60–64 years. The mortality rates of both cancers increased significantly with age, with the peak mortality rate in the 95 + age group (Fig 2A-B). In 2023, the age-specific absolute burden distribution of the four cancers was similar to that in 1990, with peaks between 50–54 years and 65–69 years (Fig 2A–C). From 1990 to 2023, the number of new cases, deaths, and DALYs of the four female-specific cancers gradually increased, and the burden on the elderly population became progressively heavier (Fig 3).

### Joinpoint regression analysis of ASIR, ASMR, ASDR for female-specific cancers in China, 1990–2023

**Fig 4:**
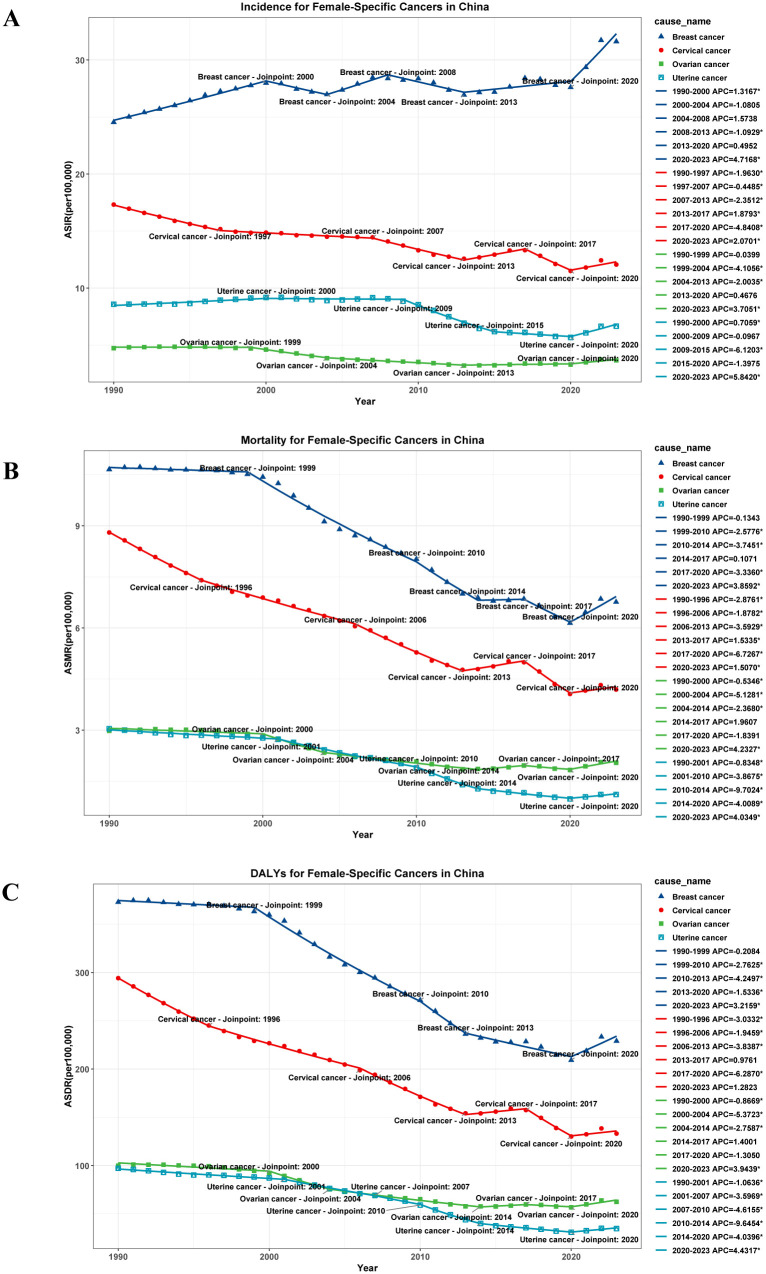
Joinpoint analysis of age-standardized rates for female-specific cancers in China, 1990–2023. (A) ASIR;(B) ASMR; (C) ASDR. “*” indicates p < 0.05. ASIR, Age-standardized incidence rate; ASMR, Age-standardized mortality rate; ASDR, Age-standardized DALY rate; DALY, disability-adjusted life year.

Joinpoint regression analysis showed that between 1990 and 2023, ASMRs and ASDRs of all female-specific cancers, and ASIRs of cervical, uterine, and ovarian cancers in China showed a downward trend; however, breast cancer ASIR increased (Fig 4). The AAPC of breast cancer ASIR was 0.81 (95% CI: 0.46 to 1.18), whereas those for cervical, ovarian, and uterine cancer ASIRs were −1.02 (95% CI: −1.31 to −0.73), −0.77 (95% CI: −1.07 to −0.46), and −0.66 (95% CI: −1.00 to −0.31), respectively. For ASMRs, the AAPCs for breast, cervical, ovarian, and uterine cancers were −1.32 (95% CI: −1.74 to −0.89), −2.17 (95% CI: −2.45 to −1.89), −1.14 (95% CI: −1.62 to −0.65), and −2.93 (95% CI: −3.21 to −2.66); corresponding AAPCs for ASDRs were −1.42 (95% CI: −1.77 to −1.06), −2.32 (95% CI: − 2.62 to −2.01), −1.41 (95% CI: −1.94 to −0.88), and −2.99 (95% CI: −3.30 to −2.68) (Fig 4, S3 Table). Although the ASRs of the four female-specific cancers had multiple inflection points over the study period, all ASRs showed an upward trend during 2020–2023 (Fig 4). During 2020–2023, the APCs of ASIR for breast, cervical, ovarian, and uterine cancers were 4.72 (95% CI: 2.83 to 6.64), 2.07 (95% CI: 0.73 to 3.43), 3.71 (95% CI: 1.94 to 5.50), and 5.84 (95% CI: 3.28 to 8.47), respectively; the corresponding APCs for ASMR were 3.86 (95% CI: 2.05 to 5.70), 1.51 (95% CI: 0.17 to 2.86), 4.23 (95% CI: 2.36 to 6.14), and 4.03 (95% CI:1.95 to 6.16); and those for ASDR were 3.22 (95% CI: 1.49 to 4.97), 1.28 (95% CI: −0.16 to 2.74), 3.94 (95% CI: 1.99 to 5.94), and 4.43 (95% CI: 2.48 to 6.42) (S3 Table).

### Age-period-cohort analysis of incidence, mortality, and DALYs for female-specific cancers in China

**Fig 5:**
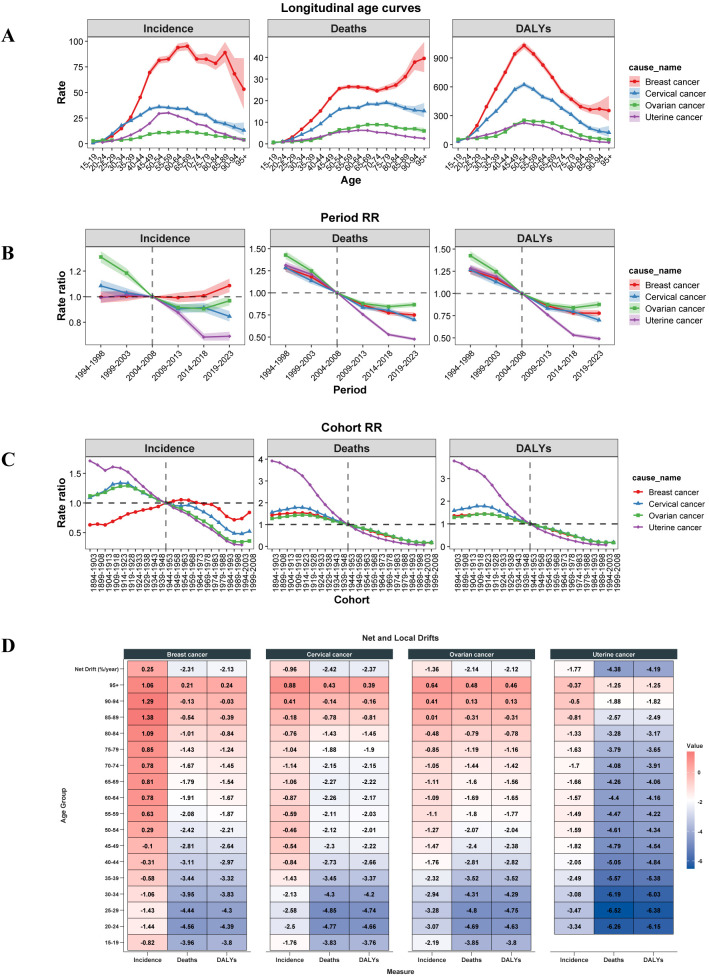
Age, period, and cohort effects on incidence, mortality, and DALYs of female-specific cancers in China. (A) Age effect; (B) Period effect; (C) Cohort effect; (D) Net and Local drifts. Shaded regions indicate the 95% confidence intervals. DALYs, disability-adjusted life years.

Age effects revealed that the age-specific incidence rates of breast cancer in Chinese women fluctuated significantly with increasing age, with two small peaks at 65–69 years and 85–89 years; the age-specific mortality rates increased with age, while age-specific DALY rates increased first and then decreased with increasing age, peaking at 50–54 years (Fig 5A). In contrast, the age-specific incidence, mortality, and DALY rates of cervical, uterine, and ovarian cancers all showed an “inverted V” trend with increasing age, with slightly different peak ages (Fig 5A). Period effects indicated that the incidence risks of breast cancer showed an upward trend, peaking in 2019–2023; in contrast, the incidence risks of cervical, uterine, and ovarian cancers decreased over time. The mortality and DALY risks of all four female-specific cancers declined over time (Fig 5B). Cohort effects revealed that with the shift to later birth cohorts, the incidence risk of breast cancer exhibited a trend of first increasing, then decreasing, and subsequently increasing again. For cohort effects on mortality and DALYs, the mortality and DALY risks of uterine cancer decreased in later birth cohorts, while those of breast, ovarian, and cervical cancers first increased slightly and then continued to decline (Fig 5C). The net drift for breast cancer incidence was > 0, while the net drifts for mortality and DALY rates were < 0—indicating that the incidence of breast cancer in Chinese women overall increased between 1990 and 2023, whereas mortality and DALY rates decreased. For cervical, ovarian, and uterine cancers, the net drifts for incidence, mortality, and DALY rates were all < 0, suggesting a downward trend for all three outcomes during the study period. Local drift highlighted that the burden of female-specific cancers in Chinese women was increasingly concentrated in the elderly population (Fig 5D).

### Decomposition analysis of incidence, mortality, and DALY numbers for female-specific cancers in China

**Fig 6:**
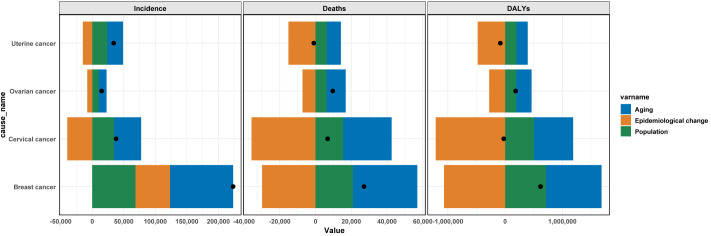
Decomposition analysis of female-specific cancer burden in China. DALYs, disability-adjusted life years.

Decomposition analysis revealed that aging and population growth were the main drivers of female-specific cancer burden in China, while epidemiological changes represented the primary mitigating factor (Fig 6). Specifically, aging increased incident cases of breast, cervical, ovarian, and uterine cancers by 44.84%, 112.91%, 84.91%, and 73.60%, respectively; corresponding increases in deaths were 133.08%, 398.71%, 111.36%, and 826.98%, and those in DALYs were 156.99%, 2897.38%, 144.50%, and 234.14%. Population growth led to increases of 30.74%, 91.61%, 67.78%, and 69.95% in incident cases of breast, cervical, ovarian, and uterine cancers, respectively; corresponding increases in deaths were 76.90%, 227.71%, 63.30%, and 663.68%, and those in DALYs were 115.50%, 2127.96%, 105.69%, and 239.70%. In contrast, epidemiological changes reduced incident cases of cervical, ovarian, and uterine cancers by 104.52%, 52.70%, and 43.35%—a mitigating effect that also extended to mortality and DALYs, with deaths from breast, cervical, ovarian, and uterine cancers decreasing by 109.98%, 526.41%, 74.66%, and 1590.66%, and DALYs declining by 172.48%, 5125.34%, 150.19%, and 573.84%, respectively (S4 Table).

### Future projections of female-specific cancer burden in China

**Fig 7:**
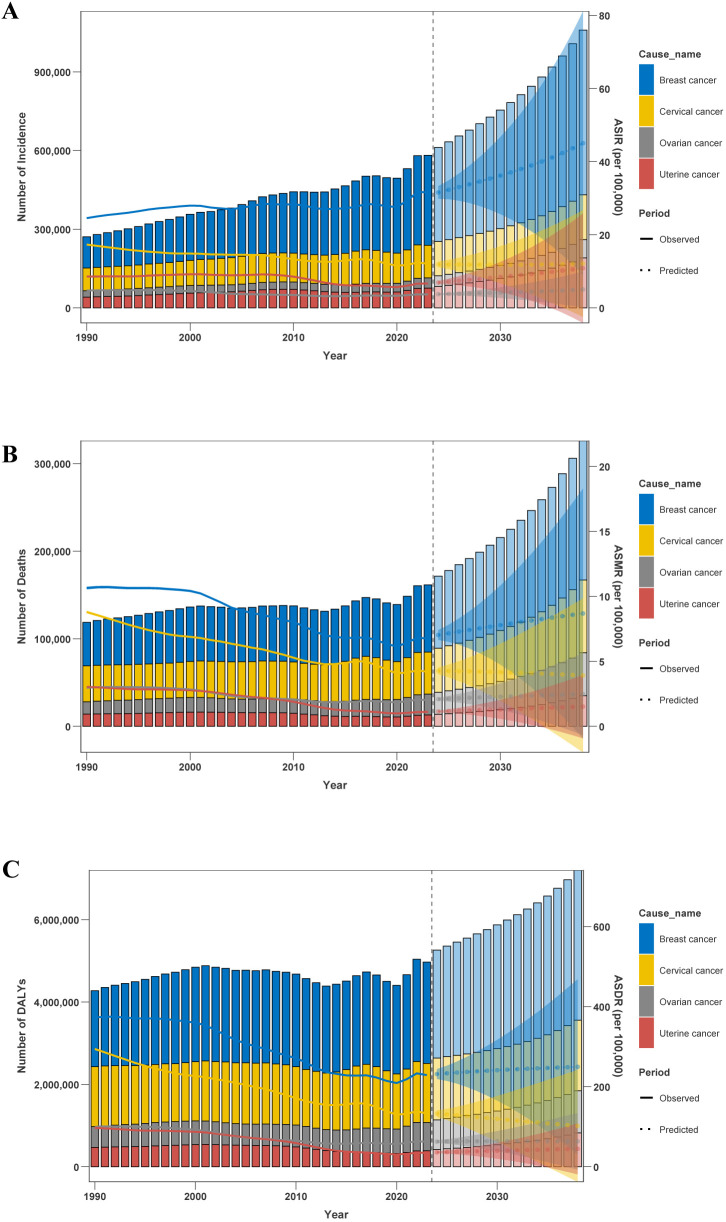
Predicted trends in the burden of female-specific cancers in China, 2024–2038. (A) Number of incidence and ASIR; (B) Number of deaths and ASMR; (C) Number of DALYs and ASDR. Shaded regions indicate the 95% uncertainty interval. ASIR, Age-standardized incidence rate; ASMR, Age-standardized mortality rate; ASDR, Age-standardized DALY rate; DALYs, disability-adjusted life years.

The BAPC model predicted that the ASRs of the four female-specific cancers in China would show different trends over the next 15 years. The ASIRs, ASMRs, and ASDRs of breast, uterine, and ovarian cancers would continue to rise; the ASIR of cervical cancer would increase slightly, while its ASMR and ASDRs would continue to decline (Fig 7). For absolute burden, the number of new cases, deaths, and DALYs of the four female-specific cancers would all show an upward trend over the next 15 years. The number of new breast cancer cases would be projected to increase from 342,725 in 2023–627,816 in 2038, a rise of 83.18%; the number of deaths would be expected to increase from 76,736 in 2023–159,000 in 2038, an increase of 107.20%; and the number of DALYs would be predicted to increase from 2,463,392 in 2023–3,640,689 in 2038, a growth of 47.79%. For cervical cancer, the predicted increases in new cases, deaths, and DALYs would be 37.51%, 73.50%, and 19.46%, respectively; for ovarian cancer, those would be 78.55%, 107.31%, and 48.79%; and for uterine cancer, the increases would be 153.81%, 169.37%, and 112.24%, respectively (Fig 7, S5 Table).

## Discussion

This study conducted a comprehensive assessment of the burden of female-specific cancers in China, reporting the long-term trends of these cancers from 1990 to 2023 based on the updated GBD 2023 database. Among the ASIRs, ASMRs, and ASDRs of the four female-specific cancers, all trended downward except for the ASIR of breast cancer—highlighting that China has made substantial progress in the prevention, control, diagnosis, and treatment of breast cancer and other gynecological cancers over the past 34 years. However, the sustained increase in absolute burden indicates that these cancers remain major public health factors threatening the health of Chinese women, underscoring an urgent need to improve Chinese women’s awareness of these cancers and their participation in early screening.

The ASIR of breast cancer increased during the study period; potential driving factors included lifestyle changes—such as reduced physical activity and excessive intake of high-fat and high-protein diets—and reproductive factors, such as decreased breastfeeding rates, delayed childbirth, and earlier menarche [35,36]. Decomposition analysis results revealed that epidemiological changes drove the increase in breast cancer incidence, primarily due to the popularization of screening, which significantly improved the detection rate of early-stage breast cancer. China launched the Rural Women’s Breast Cancer Screening Program in 2009, providing mammography or breast ultrasound examinations for high-risk female groups with a gradual increase in coverage, reaching 31% among women aged 35–64 years during 2018–2019 [37]. The age-period-cohort model revealed that the local drift of breast cancer incidence among women aged 50 years and older was > 0, indicating that the incidence rate in this population had increased over the past 34 years. The main reasons for the rising incidence in this group were decreased estrogen and progesterone secretion and increased cancer susceptibility associated with aging [6]. The ASMR and ASDR showed a significant downward trend over the past 34 years, largely attributable to medical advances—continuous progress in treatment methods greatly improved the prognosis and quality of life of breast cancer patients [5]. The BAPC model predicted that the absolute burden of breast cancer would continue to increase over the next 15 years. Targeted public health interventions are warranted in the future to mitigate this burden. First, health education should be implemented at the community or township level to advocate healthy lifestyles—such as reducing high-fat intake and increasing physical activity—popularize self-breast examination, and enhance public awareness of early breast cancer screening; women who feel breast masses during self-examination should seek timely medical attention for further diagnosis and treatment. Second, breast cancer screening strategies should be improved by incorporating breast ultrasound into routine physical examinations for women and shortening the screening interval for high-risk groups. Third, disciplinary development should be strengthened, with breast surgery, oncology, and other cancer-related departments established in county-level hospitals, and professional talents recruited to improve the diagnostic and therapeutic capabilities of breast cancer in these hospitals—this will enable more rural breast cancer patients to receive professional anti-tumor treatment. Fourth, investment in medical insurance should be increased to promote the inclusion of cutting-edge anti-tumor drugs in the medical insurance reimbursement scope, alleviating patients’ economic burden.

Among the four female-specific cancers, the burden of cervical cancer has been significantly reduced during the study period, with its ASIR showing a marked downward trend—early screening and vaccination played a crucial role in this reduction. HPV infection is the main cause of cervical cancer, which typically progresses through four stages: HPV transmission, viral persistence, progression of infected cells to precancerous lesions, and final invasion leading to cancer [38–40]; this makes cervical cancer the only female cancer that can be primarily prevented through vaccination. In 2020, the WHO announced its strategic target known as the “70-90-70” initiative: 90% of girls receive HPV vaccination before the age of 15; 70% of women undergo screening by the ages of 35 and 45; and 90% of women diagnosed with cervical lesions receive treatment [41]. The Chinese government actively responded to this WHO-established strategic target and formulated a series of cervical cancer screening and prevention programs [42]. Despite policy formulation accelerating the HPV vaccination coverage rate among Chinese women, the HPV vaccination coverage rate in China has not yet reached the level set by the WHO. A study on HPV vaccination coverage in China showed that the first-dose coverage among girls aged 9–14 years was 4.00%, and the third-dose coverage was 0.31%—far below the target established by the WHO [43]. Inequalities in economic development were also a key factor affecting vaccination coverage; HPV vaccines in China have not been included in the free vaccination program, resulting in low vaccination coverage in less developed regions [44]. Our study observed a downward trend in the ASDR and ASMR of cervical cancer; the potential reason was that advances in surgical and radiotherapy techniques greatly improved the prognosis and quality of life of patients with advanced cervical cancer [45]. This study predicted that by 2038, the number of new cervical cancer cases in China will be 171,453, the number of deaths will be 83,060, and the number of DALYs will be 1,712,473—these data indicate that cervical cancer will still pose a serious threat to the health of Chinese women in the future. In the future, policymakers should accelerate the inclusion of HPV vaccines into the national free vaccination program, further expand the age range for vaccination, and promptly implement vaccination plans in less developed regions. Targeted health education should be carried out to enhance public awareness of HPV infection and cervical cancer prevention and control, strengthen early screening efforts for cervical cancer among eligible women, and further reduce the burden of cervical cancer.

This study found that although the ASIRs of uterine and ovarian cancers in China showed a slight downward trend during the study period, the sustained increase in new cases highlighted the substantial challenges these cancers pose to the health of Chinese women. A high body mass index (BMI) is recognized as one of the risk factors for uterine and ovarian cancers [46]. An epidemiological study on obesity among Chinese adults reported that the obesity rate among Chinese adults exceeded 16% between 2015 and 2019 [47]. Hormonal abnormalities, insulin resistance, and inflammatory factor secretion induced by obesity create a favorable microenvironment for tumor initiation and progression [48]. Delayed marriage and childbearing, as well as reduced parity associated with China’s family planning policies, may have further increased the burden of ovarian cancer [49]. Decomposition analysis indicated that population aging was the main driving factor for the increase in new cases of uterine and ovarian cancers. With advancing age, the decline in DNA repair capacity makes cells more susceptible to gene mutations and chromosomal instability, thereby increasing the risk of tumorigenesis [50]. Hypertension and diabetes were common chronic diseases in the elderly, and existing studies confirmed that these conditions are among the risk factors for uterine and ovarian cancers [51,52]. In the future, efforts should be made to strengthen the coordinated management of chronic diseases and weight management in middle-aged and elderly women, incorporating modifiable risk factors such as obesity, diabetes, and hypertension into routine health monitoring. For high-risk groups, additional screening items such as transvaginal ultrasound and tumor markers should be included in health check-ups to improve the early diagnosis rate of uterine and ovarian cancers.

Joinpoint analysis indicated that between 2020 and 2023, the ASRs of the four female-specific cancers in China all showed an upward trend, which corresponded to the COVID-19 pandemic period. The reversal in disease burden during the pandemic may be jointly caused by physiological, socio-medical, and economic factors. Physiologically, inflammation associated with severe acute respiratory syndrome coronavirus 2 (SARS-CoV-2) infection could promote cancer cell proliferation, angiogenesis, and metastasis [53]. Studies have shown that SARS-CoV-2 infection leads to reduced numbers of CD4 + T cells, lymphocytes, and CD8 + T cells, impairing the body’s immune function and facilitating the occurrence and development of malignancies [54,55]. Several case reports have pointed out that COVID-19 infection may have led to decreased ovarian function and even premature ovarian insufficiency [56–58], a condition that is an important risk factor for cervical, uterine, and ovarian cancers [59]. From a socio-medical perspective, the pandemic exerted a substantial impact on the healthcare system: delays in cancer screening led to delayed diagnosis, while reduced access to medical care during lockdowns decreased the completion rate of cancer treatment, resulting in worse long-term prognosis [46]. Medical resources were severely diverted during the COVID-19 period, with delays in surgical and other cancer treatments [60]. A longer waiting time for treatment was positively correlated with adverse cancer prognosis [61]. Previous studies on breast and gynecological cancers have consistently shown that treatment delays have a negative impact on long-term survival rates [62–65]. From an economic perspective, the pandemic-induced economic stagnation and declining household incomes reduced the willingness to seek medical care and access to preventive care among vulnerable populations. During lockdowns, reduced physical activity and changes in dietary patterns among women further increased the incidence risk of female-specific cancers in China. The pathophysiological changes related to viral infection, delayed medical services, limited health resources, and changes in public lifestyles caused by the COVID-19 pandemic jointly drove the increase in ASRs of the four female-specific cancers, and also brought new challenges to the prevention and control of these cancers in China in the post-pandemic era.

This study has several limitations. First, the lack of pathological subtype data for the four female-specific cancers in the GBD database restricted a more refined burden assessment across subtypes. Second, the unavailability of provincial-level disease burden data limited further in-depth analyses and prevented us from exploring geographic disparities within China. Third, as a single-country study, our findings have limited generalizability to global or cross-national cancer prevention and control strategies. Fourth, COVID-19-related disruptions to clinical diagnosis and treatment, together with the redistribution of medical resources, have introduced substantial uncertainty into burden estimation; accordingly, the rising ASRs of female-specific cancers in China from 2020 to 2023 warrant prudent interpretation. Fifth, Joinpoint regression was based on point estimates of ASRs from the GBD, without incorporating 95% UIs into the variance structure of the trend model. Although this is a standard approach in trend analyses using GBD data, the uncertainty reported for APC and AAPC estimates only reflects sampling variation in the trend model, rather than the full uncertainty underlying GBD burden estimates. Sixth, BAPC projections depend on model smoothing assumptions and demographic forecasts, which assume the continuity of historical trends and may be sensitive to socioeconomic changes and public health emergencies; thus, results should be interpreted with caution.

## Conclusions

Driven by cancer screening, HPV vaccine coverage, and advances in medical technology, China has made substantial progress in preventing and controlling breast cancer and gynecological cancers over the past 34 years. Yet the increasing absolute burden, which is projected to continue rising over the next 15 years, emphasizes that these diseases are still major public health factors threatening the health of Chinese women. To mitigate this burden, targeted public health measures—including strengthening prevention, integrating medical resources, and enhancing public awareness of these cancers—should be implemented in the future to reduce the burden of these cancers.

## Supporting information

S1 Fig Case numbers and rates of female-specific cancers by age in China, 1990. (A) Incidence;(B) Deaths;(C) DALYs. Error bars and shaded regions denote the 95% uncertainty intervals. DALY, disability-adjusted life year.
(TIF)

S1 Table 2023 GBD world population age standard. (DOCX)

S2 Table International Classification of Diseases, 10th Revision (ICD-10) codes for breast, cervical, uterine, and ovarian cancers. (DOCX)

S3 Table Joinpoint analysis of age‑standardized rates for female‑specific cancers in China. AAPC, Average annual percentage change; APC, annual percentage change; CI, confidence interval; DALY, disability-adjusted life year.
(DOCX)

S4 Table Decomposition analysis of the incidence, mortality, and DALYs of female-specific cancers in China. DALYs, disability-adjusted life years.
(DOCX)

S5 Table BAPC model predictions of age-standardized rates and case numbers for female-specific cancers in China, 2024–2038. BAPC, Bayesian Age-Period-Cohort; DALYs, disability-adjusted life years.
(DOCX)
