# Supplementary figures and images for: Burden and temporal trends of female-specific cancers in China: A systematic analysis of the 2023 global burden of disease study
Source: PLoS One. 2026 Jun 10;21(6):e0351539. doi: 10.1371/journal.pone.0351539 (PMC13252721; doi:10.1371/journal.pone.0351539)

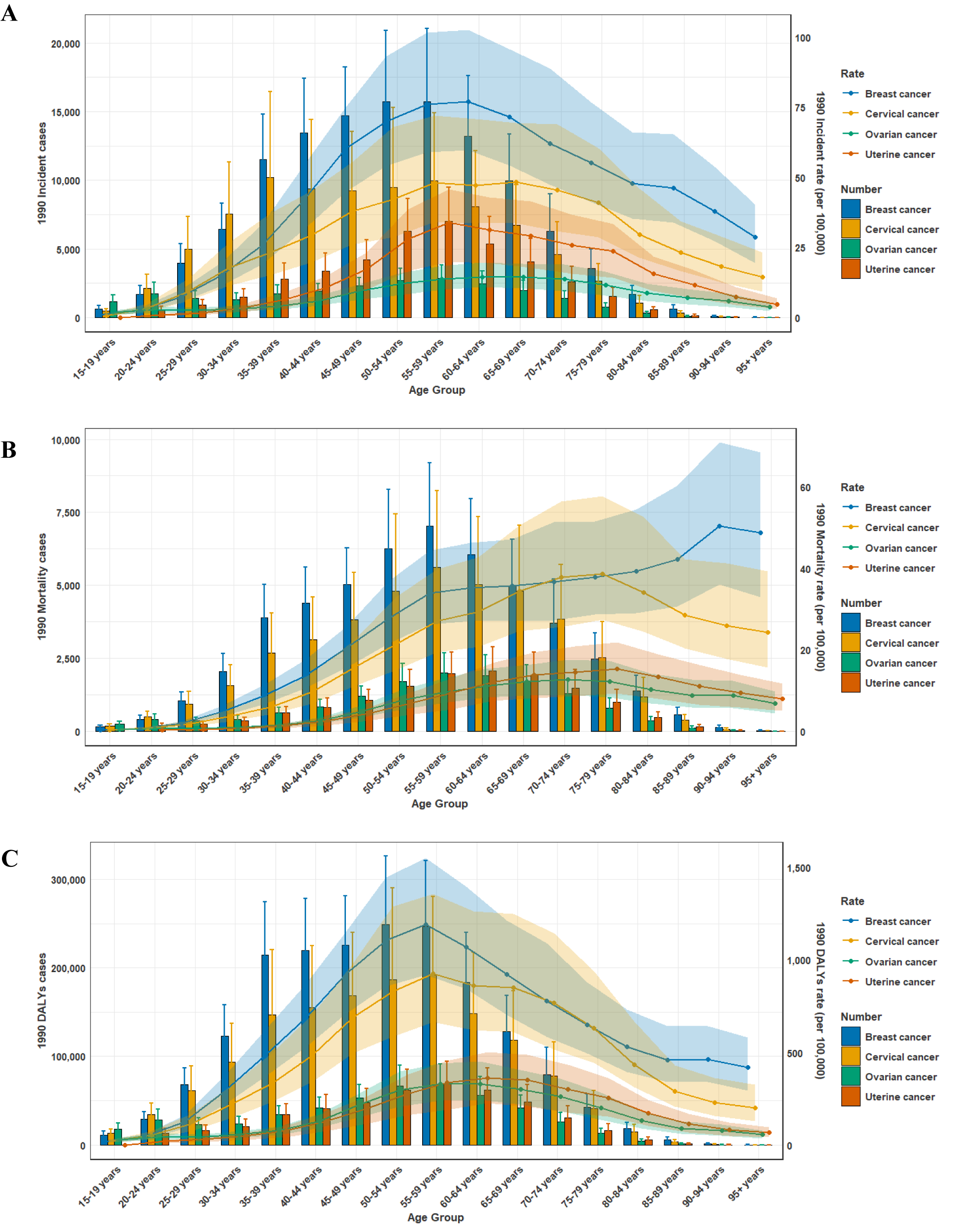

Supplement: S1 Fig — (A) Incidence;(B) Deaths;(C) DALYs. Error bars and shaded regions denote the 95% uncertainty intervals. DALY, disability-adjusted life year. (TIF) [file pone.0351539.s001.tif]
