# Supplementary material for: Burden and temporal trends of female-specific cancers in China: A systematic analysis of the 2023 global burden of disease study
Source: PLoS One. 2026 Jun 10;21(6):e0351539. doi: 10.1371/journal.pone.0351539 (PMC13252721; doi:10.1371/journal.pone.0351539)
Supplement: S1 Table — (DOCX) [file pone.0351539.s002.docx]

**S1 Table. 2023 GBD world population age standard.**

| Age Group | Standard Age Weight |
| --- | --- |
| 0-6 days | 0.000396 |
| 7-27 days | 0.001178 |
| 1-5 months | 0.008648 |
| 6-11 months | 0.010101 |
| 12-23 months | 0.020161 |
| 2-4 years | 0.059934 |
| 5-9 years | 0.096582 |
| 10-14 years | 0.089936 |
| 15-19 years | 0.082891 |
| 20-24 years | 0.078012 |
| 25-29 years | 0.075914 |
| 30-34 years | 0.073217 |
| 35-39 years | 0.068281 |
| 40-44 years | 0.061474 |
| 45-49 years | 0.055113 |
| 50-54 years | 0.049131 |
| 55-59 years | 0.043459 |
| 60-64 years | 0.036822 |
| 65-69 years | 0.029851 |
| 70-74 years | 0.022653 |
| 75-79 years | 0.015976 |
| 80-84 years | 0.010973 |
| 85-89 years | 0.006045 |
| 90-94 years | 0.002467 |
| 95+ years | 0.000785 |
