# Supplementary material for: Burden and temporal trends of female-specific cancers in China: A systematic analysis of the 2023 global burden of disease study
Source: PLoS One. 2026 Jun 10;21(6):e0351539. doi: 10.1371/journal.pone.0351539 (PMC13252721; doi:10.1371/journal.pone.0351539)
Supplement: S2 Table — (DOCX) [file pone.0351539.s003.docx]

**S2 Table. International Classification of Diseases, 10th Revision (ICD-10) codes for female breast, cervical, uterine, and ovarian cancers.**

|  | ICD-10 (incidence data) | ICD-10 (mortality data) |
| --- | --- | --- |
| Female breast cancer | C50.01, C50.011, C50.012, C50.019, C50.11, C50.111, C50.112, C50.119, C50.21, C50.211, C50.212, C50.219, C50.31, C50.311, C50.312, C50.319, C50.41, C50.411, C50.412, C50.419, C50.51, C50.511, C50.512, C50.519, C50.61, C50.611, C50.612, C50.619, C50.81, C50.811, C50.812, C50.819, C50.91, C50.911, C50.912, C50.919 | C50.01, C50.011, C50.012, C50.019, C50.11, C50.111, C50.112, C50.119, C50.21, C50.211, C50.212, C50.219, C50.31, C50.311, C50.312, C50.319, C50.41, C50.411, C50.412, C50.419, C50.51, C50.511, C50.512, C50.519, C50.61, C50.611, C50.612, C50.619, C50.81, C50.811, C50.812, C50.819, C50.91, C50.911, C50.912, C50.919, D05.01, D05.11, D05.81, D05.91 |
| Cervical cancer | C53, C53.0, C53.1, C53.3, C53.4, C53.8, C53.9 | C53, C53.0, C53.1, C53.3, C53.4, C53.8, C53.9, D06, D06.0, D06.1, D06.7, D06.9, D26.0 |
| Ovarian cancer | C56, C56.0, C56.1, C56.2, C56.4, C56.9 | C56, C56.0, C56.1, C56.2, C56.4, C56.9, D27, D27.0, D27.1, D27.9, D39.1, D39.10, D39.11, D39.12 |
| Uterine cancer | C54, C54.0, C54.1, C54.2, C54.3, C54.4, C54.8, C54.9 | C54, C54.0, C54.1, C54.2, C54.3, C54.4, C54.8, C54.9, D07.0, D07.1, D07.2, D26.1, D26.7, D26.9 |
