# Supplementary material for: Burden and temporal trends of female-specific cancers in China: A systematic analysis of the 2023 global burden of disease study
Source: PLoS One. 2026 Jun 10;21(6):e0351539. doi: 10.1371/journal.pone.0351539 (PMC13252721; doi:10.1371/journal.pone.0351539)
Supplement: S3 Table — AAPC, Average annual percentage change; APC, annual percentage change; CI, confidence interval; DALY, disability-adjusted life year. (DOCX) [file pone.0351539.s004.docx]

**S3 Table. Joinpoint analysis of age‑standardized rates for female‑specific cancers in China.**

| Age-standardized rate(per100,000) | Causes | Period | APC (95%CI) | P-Value | AAPC (95%CI) | P-Value |
| --- | --- | --- | --- | --- | --- | --- |
| Incidence | Breast cancer | 1990-2000 | 1.32 (0.99 to 1.65) | ＜0.001 | 0.81 (0.46 to 1.18) | ＜0.001 |
| Incidence | Breast cancer | 2000-2004 | -1.08 (-2.7 to 0.57) | 0.184 |  |  |
| Incidence | Breast cancer | 2004-2008 | 1.57 (-0.02 to 3.2) | 0.053 |  |  |
| Incidence | Breast cancer | 2008-2013 | -1.09 (-2.04 to -0.14) | 0.027 |  |  |
| Incidence | Breast cancer | 2013-2020 | 0.5 (-0.03 to 1.03) | 0.064 |  |  |
| Incidence | Breast cancer | 2020-2023 | 4.72 (2.83 to 6.64) | ＜0.001 |  |  |
| Incidence | Cervical cancer | 1990-1997 | -1.96 (-2.32 to -1.61) | ＜0.001 | -1.02 (-1.31 to -0.73) | ＜0.001 |
| Incidence | Cervical cancer | 1997-2007 | -0.45 (-0.68 to -0.22) | ＜0.001 |  |  |
| Incidence | Cervical cancer | 2007-2013 | -2.35 (-2.85 to -1.85) | ＜0.001 |  |  |
| Incidence | Cervical cancer | 2013-2017 | 1.88 (0.74 to 3.03) | 0.003 |  |  |
| Incidence | Cervical cancer | 2017-2020 | -4.84 (-7.06 to -2.57) | ＜0.001 |  |  |
| Incidence | Cervical cancer | 2020-2023 | 2.07 (0.73 to 3.43) | 0.005 |  |  |
| Incidence | Ovarian cancer | 1990-1999 | -0.04 (-0.5 to 0.42) | 0.858 | -0.77 (-1.07 to -0.46) | ＜0.001 |
| Incidence | Ovarian cancer | 1999-2004 | -4.11 (-5.24 to -2.95) | ＜0.001 |  |  |
| Incidence | Ovarian cancer | 2004-2013 | -2 (-2.49 to -1.51) | ＜0.001 |  |  |
| Incidence | Ovarian cancer | 2013-2020 | 0.47 (-0.14 to 1.08) | 0.127 |  |  |
| Incidence | Ovarian cancer | 2020-2023 | 3.71 (1.94 to 5.5) | ＜0.001 |  |  |
| Incidence | Uterine cancer | 1990-2000 | 0.71 (0.41 to 1) | ＜0.001 | -0.66 (-1 to -0.31) | ＜0.001 |
| Incidence | Uterine cancer | 2000-2009 | -0.1 (-0.43 to 0.24) | 0.552 |  |  |
| Incidence | Uterine cancer | 2009-2015 | -6.12 (-6.77 to -5.47) | ＜0.001 |  |  |
| Incidence | Uterine cancer | 2015-2020 | -1.4 (-2.91 to 0.13) | 0.071 |  |  |
| Incidence | Uterine cancer | 2020-2023 | 5.84 (3.28 to 8.47) | ＜0.001 |  |  |
| Deaths | Breast cancer | 1990-1999 | -0.13 (-0.54 to 0.28) | 0.499 | -1.32 (-1.74 to -0.89) | ＜0.001 |
| Deaths | Breast cancer | 1999-2010 | -2.58 (-2.82 to -2.34) | ＜0.001 |  |  |
| Deaths | Breast cancer | 2010-2014 | -3.75 (-5.14 to -2.33) | ＜0.001 |  |  |
| Deaths | Breast cancer | 2014-2017 | 0.11 (-2.82 to 3.12) | 0.94 |  |  |
| Deaths | Breast cancer | 2017-2020 | -3.34 (-6.1 to -0.49) | 0.025 |  |  |
| Deaths | Breast cancer | 2020-2023 | 3.86 (2.05 to 5.7) | ＜0.001 |  |  |
| Deaths | Cervical cancer | 1990-1996 | -2.88 (-3.34 to -2.41) | ＜0.001 | -2.17 (-2.45 to -1.89) | ＜0.001 |
| Deaths | Cervical cancer | 1996-2006 | -1.88 (-2.11 to -1.65) | ＜0.001 |  |  |
| Deaths | Cervical cancer | 2006-2013 | -3.59 (-3.97 to -3.22) | ＜0.001 |  |  |
| Deaths | Cervical cancer | 2013-2017 | 1.53 (0.47 to 2.61) | 0.007 |  |  |
| Deaths | Cervical cancer | 2017-2020 | -6.73 (-8.91 to -4.5) | ＜0.001 |  |  |
| Deaths | Cervical cancer | 2020-2023 | 1.51 (0.17 to 2.86) | 0.029 |  |  |
| Deaths | Ovarian cancer | 1990-2000 | -0.53 (-0.91 to -0.16) | 0.008 | -1.14 (-1.62 to -0.65) | ＜0.001 |
| Deaths | Ovarian cancer | 2000-2004 | -5.13 (-6.82 to -3.41) | ＜0.001 |  |  |
| Deaths | Ovarian cancer | 2004-2014 | -2.37 (-2.76 to -1.97) | ＜0.001 |  |  |
| Deaths | Ovarian cancer | 2014-2017 | 1.96 (-1.48 to 5.52) | 0.249 |  |  |
| Deaths | Ovarian cancer | 2017-2020 | -1.84 (-4.76 to 1.17) | 0.212 |  |  |
| Deaths | Ovarian cancer | 2020-2023 | 4.23 (2.36 to 6.14) | ＜0.001 |  |  |
| Deaths | Uterine cancer | 1990-2001 | -0.83 (-1.05 to -0.61) | ＜0.001 | -2.93 (-3.21 to -2.66) | ＜0.001 |
| Deaths | Uterine cancer | 2001-2010 | -3.87 (-4.14 to -3.6) | ＜0.001 |  |  |
| Deaths | Uterine cancer | 2010-2014 | -9.7 (-10.77 to -8.62) | ＜0.001 |  |  |
| Deaths | Uterine cancer | 2014-2020 | -4.01 (-4.82 to -3.19) | ＜0.001 |  |  |
| Deaths | Uterine cancer | 2020-2023 | 4.03 (1.95 to 6.16) | ＜0.001 |  |  |
| DALYs | Breast cancer | 1990-1999 | -0.21 (-0.64 to 0.23) | 0.331 | -1.42 (-1.77 to -1.06) | ＜0.001 |
| DALYs | Breast cancer | 1999-2010 | -2.76 (-3.01 to -2.51) | ＜0.001 |  |  |
| DALYs | Breast cancer | 2010-2013 | -4.25 (-7.38 to -1.02) | 0.013 |  |  |
| DALYs | Breast cancer | 2013-2020 | -1.53 (-2.04 to -1.03) | ＜0.001 |  |  |
| DALYs | Breast cancer | 2020-2023 | 3.22 (1.49 to 4.97) | ＜0.001 |  |  |
| DALYs | Cervical cancer | 1990-1996 | -3.03 (-3.53 to -2.54) | ＜0.001 | -2.32 (-2.62 to -2.01) | ＜0.001 |
| DALYs | Cervical cancer | 1996-2006 | -1.95 (-2.19 to -1.71) | ＜0.001 |  |  |
| DALYs | Cervical cancer | 2006-2013 | -3.84 (-4.23 to -3.45) | ＜0.001 |  |  |
| DALYs | Cervical cancer | 2013-2017 | 0.98 (-0.21 to 2.18) | 0.102 |  |  |
| DALYs | Cervical cancer | 2017-2020 | -6.29 (-8.7 to -3.81) | ＜0.001 |  |  |
| DALYs | Cervical cancer | 2020-2023 | 1.28 (-0.16 to 2.74) | 0.077538 |  |  |
| DALYs | Ovarian cancer | 1990-2000 | -0.87 (-1.26 to -0.47) | ＜0.001 | -1.41 (-1.94 to -0.88) | ＜0.001 |
| DALYs | Ovarian cancer | 2000-2004 | -5.37 (-7.15 to -3.56) | ＜0.001 |  |  |
| DALYs | Ovarian cancer | 2004-2014 | -2.76 (-3.19 to -2.33) | ＜0.001 |  |  |
| DALYs | Ovarian cancer | 2014-2017 | 1.4 (-2.39 to 5.33) | 0.452 |  |  |
| DALYs | Ovarian cancer | 2017-2020 | -1.3 (-4.7 to 2.21) | 0.44 |  |  |
| DALYs | Ovarian cancer | 2020-2023 | 3.94 (1.99 to 5.94) | ＜0.001 |  |  |
| DALYs | Uterine cancer | 1990-2001 | -1.06 (-1.27 to -0.86) | ＜0.001 | -2.99 (-3.3 to -2.68) | ＜0.001 |
| DALYs | Uterine cancer | 2001-2007 | -3.6 (-4.13 to -3.06) | ＜0.001 |  |  |
| DALYs | Uterine cancer | 2007-2010 | -4.62 (-6.62 to -2.56) | ＜0.001 |  |  |
| DALYs | Uterine cancer | 2010-2014 | -9.65 (-10.62 to -8.66) | ＜0.001 |  |  |
| DALYs | Uterine cancer | 2014-2020 | -4.04 (-4.81 to -3.26) | ＜0.001 |  |  |
| DALYs | Uterine cancer | 2020-2023 | 4.43 (2.48 to 6.42) | ＜0.001 |  |  |

AAPC, Average annual percentage change; APC, annual percentage change; CI, confidence interval; DALY, disability-adjusted life year.
