# Supplementary material for: Burden and temporal trends of female-specific cancers in China: A systematic analysis of the 2023 global burden of disease study
Source: PLoS One. 2026 Jun 10;21(6):e0351539. doi: 10.1371/journal.pone.0351539 (PMC13252721; doi:10.1371/journal.pone.0351539)
Supplement: S4 Table — DALYs, disability-adjusted life years. (DOCX) [file pone.0351539.s005.docx]

**S4 Table. Decomposition analysis of the incidence, mortality, and DALYs of female-specific cancers in China.**

| measure | Causes | overll_difference | Aging | Population | Epidemiological change |
| --- | --- | --- | --- | --- | --- |
| Incidence | Breast cancer | 223407.65 | 100184.5（44.84%） | 68678.83（30.74%） | 54544.31（24.41%） |
| Incidence | Cervical cancer | 37878.18 | 42767.96（112.91%） | 34699.73（91.61%） | -39589.51（-104.52%） |
| Incidence | Ovarian cancer | 14878.44 | 12633.42（84.91%） | 10085.22（67.78%） | -7840.2（-52.7%） |
| Incidence | Uterine cancer | 34006.02 | 25028.91（73.6%） | 23787.48（69.95%） | -14810.37（-43.55%） |
| Deaths | Breast cancer | 27120.32 | 36092.16（133.08%） | 20854.79（76.9%） | -29826.62（-109.98%） |
| Deaths | Cervical cancer | 6796.44 | 27097.9（398.71%） | 15476.02（227.71%） | -35777.47（-526.41%） |
| Deaths | Ovarian cancer | 9673.18 | 10772.16（111.36%） | 6122.88（63.3%） | -7221.85（-74.66%） |
| Deaths | Uterine cancer | -950.96 | 7864.27（-826.98%） | 6311.29（-663.68%） | -15126.52（1590.66%） |
| DALYs | Breast cancer | 618428.91 | 970855.71（156.99%） | 714268.57（115.5%） | -1066695.36（-172.48%） |
| DALYs | Cervical cancer | -23647.21 | 685150.15（-2897.38%） | 503203.53（-2127.96%） | -1212000.89（5125.34%） |
| DALYs | Ovarian cancer | 184745.83 | 266951.69（144.5%） | 195262.01（105.69%） | -277467.87（-150.19%） |
| DALYs | Uterine cancer | -83501.74 | 195508.24（-234.14%） | 200153.45（-239.7%） | -479163.43（573.84%） |

DALYs, disability-adjusted life years.
