# Supplementary material for: Burden and temporal trends of female-specific cancers in China: A systematic analysis of the 2023 global burden of disease study
Source: PLoS One. 2026 Jun 10;21(6):e0351539. doi: 10.1371/journal.pone.0351539 (PMC13252721; doi:10.1371/journal.pone.0351539)
Supplement: S5 Table — BAPC, Bayesian Age-Period-Cohort; DALYs, disability-adjusted life years. (DOCX) [file pone.0351539.s006.docx]

**S5 Table.** **BAPC model predictions of age-standardized rates and case numbers for female-specific cancers in China, 2024–2038.**

| Year | Measure | ASR (per 100,000)/ (95% UI) | | | | Number | | |  |
| --- | --- | --- | --- | --- | --- | --- | --- | --- | --- |
|  |  | Breast cancer | Cervical cancer | Ovarian cancer | Uterine cancer | Breast cancer | Cervical cancer | Ovarian cancer | Uterine cancer |
| 2024 | Incidence | 31.57 (29.85-33.29) | 11.91 (11.12-12.7) | 3.73 (3.54-3.93) | 6.92 (6.48-7.36) | 358765 | 130376 | 41146 | 81283 |
| 2025 | Incidence | 32.3 (29.74-34.87) | 11.88 (10.62-13.14) | 3.8 (3.48-4.13) | 7.17 (6.38-7.96) | 373087 | 131867 | 42555 | 85741 |
| 2026 | Incidence | 33.04 (29.34-36.74) | 11.86 (10-13.71) | 3.87 (3.38-4.36) | 7.42 (6.17-8.68) | 387697 | 133325 | 43973 | 90359 |
| 2027 | Incidence | 33.79 (28.71-38.87) | 11.83 (9.29-14.38) | 3.94 (3.25-4.63) | 7.68 (5.86-9.49) | 402699 | 134836 | 45418 | 95202 |
| 2028 | Incidence | 34.56 (27.88-41.24) | 11.81 (8.5-15.13) | 4.01 (3.1-4.93) | 7.93 (5.46-10.41) | 418362 | 136482 | 46926 | 100334 |
| 2029 | Incidence | 35.36 (26.86-43.86) | 11.8 (7.65-15.95) | 4.09 (2.92-5.26) | 8.19 (4.96-11.43) | 434789 | 138298 | 48515 | 105806 |
| 2030 | Incidence | 36.19 (25.66-46.72) | 11.79 (6.73-16.84) | 4.16 (2.71-5.62) | 8.46 (4.37-12.55) | 451980 | 140334 | 50205 | 111708 |
| 2031 | Incidence | 37.06 (24.28-49.85) | 11.79 (5.77-17.81) | 4.25 (2.48-6.01) | 8.73 (3.68-13.78) | 469955 | 142618 | 52013 | 118135 |
| 2032 | Incidence | 37.99 (22.72-53.26) | 11.8 (4.75-18.84) | 4.33 (2.23-6.44) | 9.01 (2.9-15.11) | 488609 | 145165 | 53941 | 125180 |
| 2033 | Incidence | 38.97 (20.97-56.97) | 11.82 (3.68-19.96) | 4.43 (1.95-6.91) | 9.29 (2.01-16.57) | 508137 | 148052 | 55999 | 132978 |
| 2034 | Incidence | 40.02 (19.02-61.02) | 11.85 (2.55-21.14) | 4.53 (1.64-7.41) | 9.58 (1.02-18.14) | 528939 | 151391 | 58248 | 141698 |
| 2035 | Incidence | 41.15 (16.87-65.43) | 11.89 (1.38-22.41) | 4.64 (1.3-7.97) | 9.88 (-0.09-19.85) | 551108 | 155262 | 60699 | 151516 |
| 2036 | Incidence | 42.35 (14.48-70.23) | 11.95 (0.15-23.76) | 4.75 (0.94-8.57) | 10.19 (-1.31-21.69) | 574850 | 159810 | 63385 | 162682 |
| 2037 | Incidence | 43.65 (11.85-75.46) | 12.03 (-1.15-25.21) | 4.88 (0.54-9.22) | 10.51 (-2.65-23.68) | 600387 | 165177 | 66338 | 175493 |
| 2038 | Incidence | 45.04 (8.94-81.14) | 12.12 (-2.51-26.75) | 5.02 (0.1-9.93) | 10.85 (-4.13-25.82) | 627816 | 171453 | 69578 | 190332 |
| 2024 | Deaths | 7.03 (6.65-7.41) | 4.28 (4.01-4.54) | 2.09 (1.98-2.21) | 1.14 (1.07-1.22) | 82349 | 50395 | 25001 | 13737 |
| 2025 | Deaths | 7.16 (6.51-7.81) | 4.26 (3.77-4.75) | 2.13 (1.92-2.34) | 1.17 (1.03-1.31) | 85920 | 51487 | 26067 | 14458 |
| 2026 | Deaths | 7.29 (6.28-8.3) | 4.25 (3.49-5.01) | 2.16 (1.83-2.49) | 1.2 (0.97-1.43) | 89611 | 52616 | 27166 | 15221 |
| 2027 | Deaths | 7.42 (5.99-8.85) | 4.23 (3.16-5.3) | 2.19 (1.72-2.67) | 1.23 (0.9-1.56) | 93414 | 53801 | 28302 | 16035 |
| 2028 | Deaths | 7.55 (5.64-9.46) | 4.21 (2.79-5.63) | 2.22 (1.59-2.86) | 1.25 (0.8-1.7) | 97404 | 55095 | 29497 | 16921 |
| 2029 | Deaths | 7.67 (5.22-10.12) | 4.19 (2.4-5.98) | 2.25 (1.44-3.07) | 1.28 (0.7-1.86) | 101678 | 56561 | 30787 | 17907 |
| 2030 | Deaths | 7.79 (4.75-10.83) | 4.17 (1.98-6.35) | 2.28 (1.27-3.3) | 1.31 (0.58-2.04) | 106246 | 58207 | 32178 | 19003 |
| 2031 | Deaths | 7.91 (4.23-11.6) | 4.14 (1.54-6.75) | 2.31 (1.08-3.54) | 1.33 (0.44-2.23) | 111117 | 60051 | 33677 | 20220 |
| 2032 | Deaths | 8.03 (3.65-12.41) | 4.12 (1.07-7.16) | 2.34 (0.88-3.8) | 1.36 (0.29-2.43) | 116292 | 62104 | 35285 | 21568 |
| 2033 | Deaths | 8.14 (3.01-13.28) | 4.09 (0.59-7.58) | 2.36 (0.66-4.07) | 1.39 (0.13-2.65) | 121862 | 64428 | 37024 | 23078 |
| 2034 | Deaths | 8.26 (2.32-14.19) | 4.06 (0.1-8.02) | 2.39 (0.42-4.36) | 1.41 (-0.05-2.88) | 127973 | 67133 | 38953 | 24806 |
| 2035 | Deaths | 8.37 (1.59-15.15) | 4.02 (-0.41-8.46) | 2.41 (0.17-4.65) | 1.44 (-0.25-3.13) | 134676 | 70271 | 41093 | 26797 |
| 2036 | Deaths | 8.48 (0.8-16.16) | 3.99 (-0.94-8.92) | 2.43 (-0.1-4.97) | 1.47 (-0.46-3.39) | 142026 | 73910 | 43472 | 29109 |
| 2037 | Deaths | 8.58 (-0.05-17.21) | 3.96 (-1.47-9.38) | 2.45 (-0.38-5.29) | 1.49 (-0.68-3.67) | 150071 | 78124 | 46112 | 31807 |
| 2038 | Deaths | 8.69 (-0.94-18.31) | 3.92 (-2.01-9.85) | 2.47 (-0.68-5.63) | 1.52 (-0.92-3.96) | 159000 | 83060 | 49070 | 34989 |
| 2024 | DALYs | 231.59 (219.27-243.91) | 132.8 (124.66-140.94) | 62.88 (59.56-66.2) | 35.95 (33.7-38.19) | 2621428 | 1500891 | 719832 | 418613 |
| 2025 | DALYs | 233.49 (214.65-252.32) | 130.68 (116.72-144.63) | 63.19 (57.52-68.86) | 36.62 (32.57-40.68) | 2685740 | 1500907 | 736270 | 435252 |
| 2026 | DALYs | 235.32 (207.9-262.74) | 128.55 (107.41-149.69) | 63.48 (54.8-72.15) | 37.3 (30.92-43.69) | 2749780 | 1500969 | 752774 | 452591 |
| 2027 | DALYs | 237.01 (199.44-274.58) | 126.39 (97.17-155.6) | 63.71 (51.55-75.87) | 37.98 (28.82-47.14) | 2813413 | 1501636 | 769420 | 470702 |
| 2028 | DALYs | 238.53 (189.51-287.55) | 124.17 (86.24-162.09) | 63.88 (47.84-79.92) | 38.63 (26.3-50.96) | 2876896 | 1503323 | 786318 | 489817 |
| 2029 | DALYs | 239.93 (178.3-301.57) | 121.92 (74.77-169.06) | 64.01 (43.73-84.29) | 39.28 (23.4-55.16) | 2941402 | 1506898 | 803841 | 510382 |
| 2030 | DALYs | 241.29 (165.96-316.62) | 119.68 (62.93-176.42) | 64.11 (39.27-88.95) | 39.92 (20.12-59.72) | 3007515 | 1512837 | 822195 | 532646 |
| 2031 | DALYs | 242.6 (152.56-332.65) | 117.45 (50.8-184.1) | 64.18 (34.48-93.89) | 40.55 (16.47-64.64) | 3075382 | 1521577 | 841519 | 556876 |
| 2032 | DALYs | 243.82 (138.13-349.51) | 115.23 (38.46-191.99) | 64.21 (29.38-99.05) | 41.18 (12.46-69.9) | 3144761 | 1533332 | 861849 | 583256 |
| 2033 | DALYs | 244.89 (122.69-367.09) | 112.98 (25.98-199.98) | 64.19 (23.99-104.38) | 41.79 (8.1-75.48) | 3216014 | 1548459 | 883170 | 612105 |
| 2034 | DALYs | 245.89 (106.32-385.45) | 110.74 (13.41-208.07) | 64.12 (18.34-109.91) | 42.38 (3.37-81.39) | 3290908 | 1568356 | 906195 | 644198 |
| 2035 | DALYs | 246.9 (89.1-404.7) | 108.55 (0.82-216.27) | 64.05 (12.46-115.64) | 42.97 (-1.7-87.64) | 3370471 | 1593908 | 931279 | 680351 |
| 2036 | DALYs | 247.94 (71.05-424.83) | 106.41 (-11.76-224.57) | 63.97 (6.36-121.58) | 43.57 (-7.11-94.24) | 3455104 | 1625934 | 958647 | 721477 |
| 2037 | DALYs | 248.95 (52.17-445.73) | 104.3 (-24.29-232.9) | 63.86 (0.05-127.67) | 44.15 (-12.87-101.18) | 3544842 | 1665128 | 988393 | 768554 |
| 2038 | DALYs | 249.88 (32.48-467.29) | 102.21 (-36.74-241.17) | 63.72 (-6.44-133.88) | 44.72 (-18.98-108.43) | 3640689 | 1712473 | 1020802 | 822593 |

ASRs, Age-standardized rates; BAPC, Bayesian Age-Period-Cohort, DALYs, disability-adjusted life years.
